# Mechanical, Structural, and Biological Characteristics of Polylactide/Wollastonite 3D Printed Scaffolds

**DOI:** 10.3390/polym14193932

**Published:** 2022-09-20

**Authors:** Rajan Choudhary, Inna Bulygina, Vladislav Lvov, Anna Zimina, Sergey Zhirnov, Evgeny Kolesnikov, Denis Leybo, Natalya Anisimova, Mikhail Kiselevskiy, Maria Kirsanova, Fedor Senatov

**Affiliations:** 1 Rudolfs Cimdins Riga Biomaterials Innovations and Development Centre of RTU, Faculty of Materials Science and Applied Chemistry, Institute of General Chemical Engineering, Riga Technical University, Kipsala Street 6A, LV-1048 Riga, Latvia; 2 Baltic Biomaterials Centre of Excellence, Headquarters at Riga Technical University, Kipsala Street 6A, LV-1048 Riga, Latvia; 3 Center for Biomedical Engineering, National University of Science and Technology “MISIS”, Leninskiy pr., 6s7, 119049 Moscow, Russia; 4 N. N. Blokhin National Medical Research Centre of Oncology of the Health Ministry of Russia (N. N. Blokhin NMRCO), Kashirskoye sh. 24, 115478 Moscow, Russia; 5 Advanced Imaging Core Facility, Skolkovo Institute of Science and Technology, 3 Nobel Str., 121205 Moscow, Russia

**Keywords:** polymer-matrix composites (PMCs), porosity/voids, finite element analysis (FEA), scanning/transmission electron microscopy (STEM), 3D printing

## Abstract

The present work aimed to study the synergistic response of bioresorbable polylactide/bioactive wollastonite scaffolds towards mechanical stability, mesenchymal stromal cell colonization, and antibacterial activity in the physiological environment. Wollastonite was synthesized at 800 °C within 2 h by sol-gel combustion method. The surface area was found to be 1.51 m^2^/g, and Transmission Electron Microscopy (TEM) micrographs indicated the presence of porous structures. Fused deposition modeling was used to prepare 3D-printed polylactide/wollastonite and polylactide/hydroxyapatite scaffolds. Scanning Electron Microscopy (SEM) micrographs confirmed the interconnected porous structure and complex geometry of the scaffolds. The addition of wollastonite decreased the contact angle of the scaffolds. The mechanical testing of scaffolds examined by computational simulation, as well as machine testing, revealed their non-load-bearing capacity. The chemical constituent of the scaffolds was found to influence the attachment response of different cells on their surface. The incorporation of wollastonite effectively reduced live bacterial attachment, whereas the colonization of mesenchymal cells was improved. This observation confirms polylactide/wollastonite scaffold possesses both bactericidal as well as cytocompatible properties. Thus, the risk of peri-implant bacterial film formation can be prevented, and the biological fixation of the scaffold at the defect site can be enhanced by utilizing these composites.

## 1. Introduction

Pore interconnectivity and controllable porosity are desirable properties of bone scaffolds [1]. The fabrication of scaffolds with such properties is still a major challenge in the bioengineering field. Traditional fabrication methods (freeze-drying, salt leaching, foaming, polymer-sponge) partially fulfill these requirements [2]. The utilization of organic solvents affects cell viability, and sample loss during fabrication is the shortcoming of these traditional methods [3]. Moreover, the desired internal architecture, porosity, shape, and geometry of the scaffold are still impossible to achieve for these methods. With the advancements in additive manufacturing technology, the preparation of biomimetic scaffolds having structural complexities similar to that of host tissues has become a reality [4,5,6,7]. Biomedical engineers are employing additive manufacturing rapid prototyping technology for the development of scaffolds with a controllable structure for tissue regeneration [8,9,10,11].

The most widespread 3D printing technique is fused deposition modeling (FDM). FDM, in comparison with other techniques, is rapid, cost-effective, and a readily available process [12,13,14]. Literature findings have indicated FDM as a simple and versatile approach for the preparation of polymer-based porous scaffolds for bone tissue engineering [1] (p. 1). The demand for the development of scaffolds possessing both bioresorbability as well as bioactivity is achieved by combining polymers and bioceramics. These biomaterials have been reported as the most suitable candidates for restoring bone defects [15,16,17].

Among the bioresorbable 3D printing materials, polylactide (PLA) and its composites with hydroxyapatite (HAp) are well studied [18,19,20,21,22]. Scaffolds based on PLA/HAp composites demonstrated enhanced mineralization and osteoinduction in comparison with PLA-based scaffolds [23]. It has been found that the biomineralization and cytocompatibility of HAp can be enhanced by the incorporation of silica in HAp [24]. Similarly, 40% of bone growth was noticed in pure HAp, whereas the growth rate was increased to 60% in silica-containing HAp [25]. Thus, silicate bioceramics are predicted as an alternative to calcium phosphates.

Wollastonite (CaSiO_3_) has been found as the most explored silicate bioceramic. The biomineralization ability of wollastonite is found to be very rapid as compared to other bioactive ceramics [26,27]. Hoppe et al. (2013) emphasized the vital role of calcium as well as silicon in bone formation [28]. Recent articles also confirmed wollastonite as a potential antibacterial bioceramic [29]. Three-dimensional printing of PLA/wollastonite composites for biomedical applications is the least explored [30,31]. Moreover, few articles have reported only the mechanical, thermal, and biodegradation of composites containing PLA and wollastonite [32,33,34]. However, negligible reports were identified in the literature about studying the biocompatibility as well as the antibacterial activity of PLA/wollastonite scaffolds. Combining the bioactivity, bioresorbability, and antibacterial activity with desired porosity will assist in the development of ideal constructions for bone tissue regeneration.

Silver has been used for its antibacterial property. The therapeutic window of silver is relatively narrow, and the accumulation of antibacterial ions in the bone compromises the cytocompatibility of the biomaterial [35,36]. The proper control over concentration and the type of dopants become crucial for determining antibacterial as well as osteogenic properties simultaneously. Ideally, materials having simultaneous osteogenic potential and antibacterial ability can be extremely promising for hard tissue regeneration. A literature survey showed that silicate ceramics (wollastonite, akermanite, diopside, forsterite, etc.) and 45S5 bioglass possess reasonable antibacterial properties against clinical pathogens as compared to HAp ([29], (p. 2)) [37,38,39,40,41,42]. The in vitro/in vivo osteogenesis response of silicate ceramics indicates that they can be potential candidates for bone scaffolds [43]. Thus, exploring silicate bioceramics in combination with resorbable polymers can become an emerging research area of great interest for various biomedical applications.

In this study, the bone scaffolds based on PLA, PLA/HAp, and PLA/wollastonite were prepared using 3D printing technology. The present report demonstrates the low-temperature synthesis of wollastonite and explores the detailed structural, mechanical, and biological performance of PLA-based composites. It is known that the adhesion and intensity of the formation of peri-implant biofilm may depend on the surface properties and chemical composition of biomaterials [44]. The objective of the present study is to examine the influence of composition and surface topography on the adhesion of *Escherichia coli* (*E. coli*) and multipotent mesenchymal stromal cells (MSCs). Three-dimensional printed polylactide/wollastonite and polylactide/HAp scaffolds were used to evaluate and compare the adhesion potential of the cells and the bacteria over their surface.

## 2. Materials and Methods

Hydroxyapatite (needle-shaped particles, 90 nm, Ca/P ratio of 1.67, “Polistom”, Moscow, Russia), Polylactide (pellets, molecular weight of 110 kg/mol, Ingeo 4032D by Natureworks LLC, Minnetonka, MN, USA). Calcium Nitrate Tetrahydrate (RusHim, Moscow, Russia), Tetraethyl Orthosilicate (TEOS) (EKOS-1, Moscow, Russia, 99.99%, TU 2637-187-44493179-2014), Glycine (RusHim, Moscow, Russia), Concentrated Nitric Acid (70%, RusHim, Moscow, Russia) and deionized water was used for the synthesis of wollastonite.

### 2.1. Synthesis of Wollastonite

Wollastonite (CaSiO_3_) was synthesized by the ball mill-assisted sol-gel combustion method, and glycine was used as a fuel. An equal volume of calcium nitrate (1 M) and glycine (0.55 M) were mixed in a beaker under constant stirring. Later, 1 M TEOS was added, and formation of an aqueous transparent layer on the surface of the reaction mixture was noticed. Finally, concentrated nitric acid was used to maintain the pH at 1.7 and stirred vigorously to obtain a homogeneous mixture. This step facilitates the initiation of polycondensation and gelation process due to hydrolysis of tetraethyl orthosilicate. A transparent viscous gel-like structure was noticed after 2 days of stirring and later heated for an hour at 60 °C. The beaker was covered and kept undisturbed for 1 week of aging. The aged gel was dried in hot air oven at 110 °C. The dried gel was combusted in a preheated furnace at 350 °C for 30 min. The decomposed sample was initially calcined at 700 °C to remove residual moieties of carbon as well as nitrates. The calcined powder was ball milled (Fritsch Pulverisette, alumina balls) for 2 h (300 rpm rotational speed, and ball to powder mass ratio was 5/1) to reduce its particle size. Finally, the ball-milled sample was calcined at 800 °C for 2 h to achieve the phase purity of wollastonite.

### 2.2. Composites and Scaffolds Preparation

Twin-screw micro compounder (HAAKE MiniLab II, Thermo Fisher Scientific, Waltham, MA, USA) was employed for the preparation of PLA-based composites (20 wt. % of HAp and wollastonite (Wol)) filaments. The blends were mixed at 180 °C for 20 min, extruded, and pelletized. The filaments for 3D printing were obtained at 180 °C (PLA and PLA/HAp) and 165 °C (PLA/Wol).

**Figure 1:**
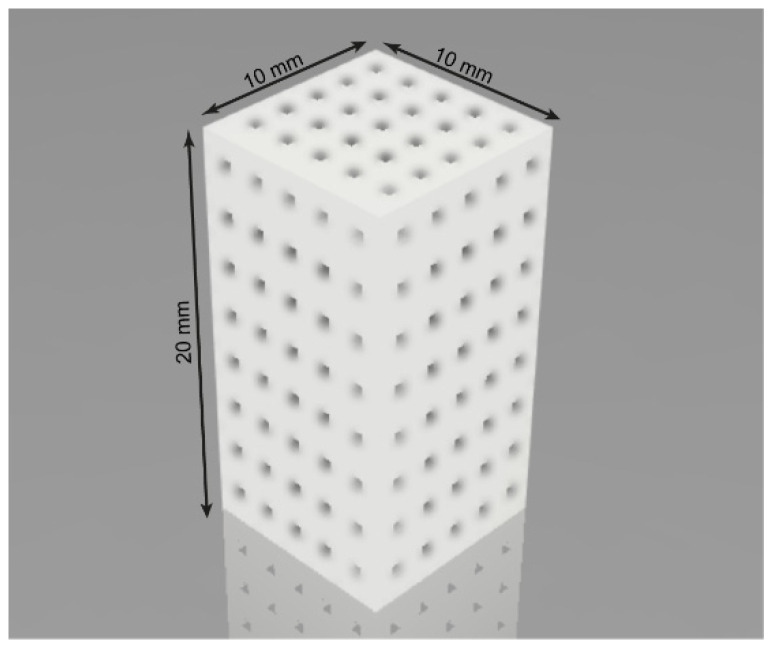
Three dimensional model of a scaffold with interconnected pores 600 µm.

The 3D model of a scaffold was prepared by using the software *Autodesk Fusion 360 2021* (Autodesk, Inc. Santa Monica, CA, USA) as shown in Figure 1. The pore size of the model was 600 μm with interconnected pores. The porosity level of the scaffolds was 19 vol. %.

The diameter of the filaments used for FDM 3D printing of the samples was 1.5± 0.3 mm. BiZon Prusa i3 Steel *PRO* (3DiY, Russia) was employed for 3D printing of the scaffolds. These scaffolds were fabricated by using optimal print settings (Table S1) gained experimentally for each filament using PrusaSlicer v.2.3.1 (Prusa Research a.s., Prague, Czech Republic).

### 2.3. Particles and Scaffolds Characterization

Thermogravimetric analysis differential scanning calorimetry (TG-DSC) of wollastonite precursor was conducted using an SDT Q600 (TA Instruments, New Castle, DE, USA). The sample was heated from 25 °C to 800 °C in an argon atmosphere with a temperature rising rate of 10 °C/min. Fourier transform infrared spectroscopy (FT-IR, Nicolet 380 spectrometer, Thermo Scientific, Waltham, MA, USA) was used to analyze the functional groups of the samples ranging from 650–4000 cm^−1^ in ATR mode. X-ray diffraction (XRD) of wollastonite and hydroxyapatite powders was measured using Ultima IV diffractometer (Rigaku, Japan) from 10° to 100° at a scanning rate of 5°/min with Co Kα radiation source (λ = 1.7902 Å). The surface morphology, microstructure, particle size, and elemental analysis of the scaffolds were characterized by scanning electron microscopy (SEM, VEGA3 TESCAN, Czech Republic, accelerating voltage of 20 kV) coupled with energy-dispersive X-ray spectroscopy (EDX, SDD-X-act Detector, Oxford Instruments Inc., Oxford, UK) in secondary electron mode. The particle morphology was studied by high-angle annular dark-field scanning transmission electron microscopy (HAADF-STEM) using Titan Themis Z transmission electron microscope (Thermo Fisher Scientific, Waltham, MA, USA) operated at 200 kV. Analysis of the adsorption–desorption isotherms was performed using Nova 1200e analyzer in nitrogen (Quantachrome Instruments, Boynton Beach, FL, USA) under relative pressure (P/P_0_) between 0.01 and 0.99.

### 2.4. Scaffolds Mechanical Testing

Universal testing machine equipped with 1 and 20 kN sensors (Zwick/Roell Z020, Zwick GmbH & Co. KG, Germany) was used to analyze the mechanical stability of the scaffolds. Additionally, to determine areas with maximum stresses (von Mises stress), equivalent strains, and possible mechanisms of destruction of the scaffold, a series of simulations of quasi-static uniaxial compression tests was carried out. The simulations of mechanical tests were performed using the Autodesk Fusion 360 2021 software with support for the study type Nonlinear Static Stress. The compressive strength and Young’s modulus (according to ASTM D695) of the scaffolds were determined by static compression tests. Rectangular scaffolds with dimensions of 10 mm × 10 mm × 20 mm (Figure S1) were prepared for this study.

### 2.5. Study of Cell Colonization and Antibacterial Properties

The hydrophilicity of the composites was evaluated by measuring the water wetting angles of non-porous PLA, PLA/HAp, and PLA/Wol specimens using the wetting angle measurement equipment EasyDrop DSA20 (KRÜSS, Germany). The contact angle was determined using the sessile drop method. The distilled water drop was 5 µL to prevent gravitational distortion of the spherical profile. Each result was evaluated as an average value of five measurements.

The model with dimensions of 10 mm × 10 mm × 3 mm was 3D printed using PLA, PLA/HAp, and PLA/Wol filaments. The scaffolds were sterilized in 70% ethanol for 24 h and dried in a sterile environment.

#### 2.5.1. MSCs Colonization

The cellular study procedures were assessed and approved by the Ethical Committee of the N. N. Blokhin NMRCO (Identification code: AAAAA-A19-119061190077-2, approval date: 12 May 2021). Mouse bone marrow MSCs were used in this work. The cell cultures collection was from the N.N. Blokhin NMRCO. The cells were treated with trypsin in the logarithmic growth phase, and Dulbecco’s Modified Eagle’s Medium (DMEM, Sigma-Aldrich, St. Louis, MO, USA) was used for washing. After washing, the cells were resuspended in a DMEM-based complete growth medium supplemented with 10% Fetal Bovine Serum (FBS), 1% penicillin/streptomycin, and 4 mM L-glutamine (PanEco, Russia) at a concentration of 620,000 cells/mL.

The surface of the scaffolds (n = 3) was seeded with 20 μL of MSCs suspension and incubated for 30 min at 37 °C in a 5% carbon dioxide atmosphere. Later, a complete growth medium (2 mL) was added to the wells with scaffolds and incubated under similar conditions for one week. The growth medium was refreshed after every two days. Finally, the scaffolds with the adhered cells were washed and stained using the Live/Dead Cell Double Staining Kit (R&D, Sigma-Aldrich, St. Louis, MO, USA). The stained scaffolds were examined by using a Lionheart FX automated microscope (BioTek, Winooski, VT, USA). 

#### 2.5.2. Antibacterial Activity

An 18 h *E. coli* culture (collection of the N.N. Blokhin NMRCO) in Mueller-Hinton broth (Pronadisa, Spain) was used to study the antibacterial activity of the scaffolds (n = 3). This study was performed according to the reported procedure [45]. In order to evaluate the antibacterial activity, fluorescent staining of samples was performed by using Live/Dead BacLight Bacterial Viability Kit for quantitative assays (Invitrogen, Waltham, MA, USA) and their subsequent study with the Spark plate reader (Tecan, San Jose, CA, USA). Stimulation of the adhesion of live *E. coli* bacteria by PLA-based materials of various compositions was estimated as the percentage of fluorescence of live bacteria attached to the sample surface relative to the initial level of bacteria fluorescence (Control). Antibacterial activity was evaluated according to the ratio of live and dead bacteria adhered to the surface of PLA-based samples. The live and dead bacteria were analyzed by Syto 9 and propidium iodide, respectively.

#### 2.5.3. Statistics

The results of statistical analysis were presented as a mean ± Standard Deviation (Mean ± SD).

## 3. Results and Discussion

### 3.1. Characterization of Wollastonite

**Figure 2:**
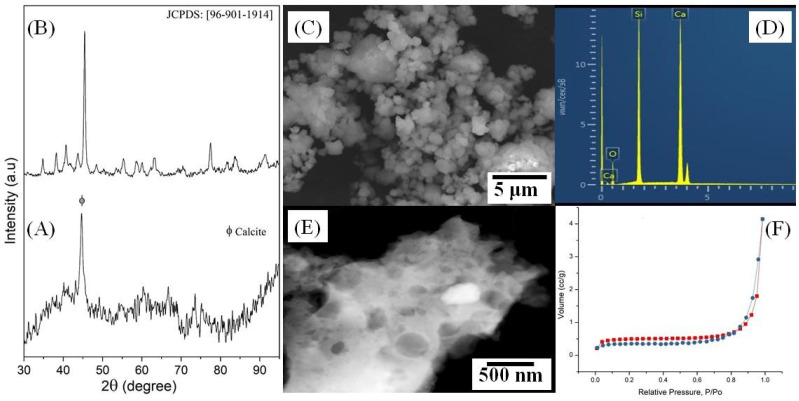
XRD patterns of combusted precursor (**A**) and wollastonite calcined at 800 °C for 2 h (**B**). SEM image (**C**); EDX spectrum (**D**) and HAADF-STEM (**E**) image of pure wollastonite. Nitrogen adsorption–desorption isotherm of pure wollastonite (**F**).

Figure S2 shows the TG-DSC thermogram of the dried wollastonite sample. FT-IR spectroscopy was used to identify the functional groups in the combusted wollastonite sample (Figure S3). Powder XRD was employed to study the phase transformation of wollastonite (Figure 2A,B). The presence of an intense peak in the XRD pattern indicates the appearance of calcite after combustion. Moreover, the surface was found to be amorphous. The ball milling of combusted sample and subsequently heating at 800 °C for 2 h lead to the synthesis of pure wollastonite. A similar XRD pattern has been reported by Lakshmi et al. at 900 °C calcined for 6 h [46]. Thus, combining ball-milling with the sol-gel combustion method assisted in preparing wollastonite within 2 h whereas a recent study took at least 6 h to achieve a similar objective ([29] (p. 2)). This report confirms wollastonite was synthesized within 2 h at 800 °C for the first time.

According to the SEM micrograph (Figure 2C), pure wollastonite is composed of agglomerated particles of arbitrary shape and size ranging from 347 to 829 nm. The typical EDX spectrum (Figure 2D) confirmed the presence of Ca, Si, and O in the sample, which satisfies the elemental composition of wollastonite (CaSiO_3_).

HAADF-STEM images indicated the presence of porous structures on the surface of wollastonite (Figure 2E). These pores are composed of different shapes and sizes. As a result, crater-like surface morphology was observed.

According to the IUPAC classification, the nitrogen adsorption–desorption curve for the wollastonite sample (Figure 2F) corresponds to type II (with a small loop in the region of 0.8–0.98 P/P_0_). This type of curve corresponds to macroporous materials, but the presence of a small loop indicates the presence of mesopores, which corroborates with HAADF-STEM images. This behavior of the material is expectable when using high processing temperatures, leading to agglomeration and destruction of mesopore walls [47]. The average pore diameter was found to be 2.0 nm, with a total mesopore volume of 0.007 cm^3^/g. The specific surface area, according to BET, is 1.51 m^2^/g.

### 3.2. Characterization of HAp

FT-IR spectroscopy was used to detect the presence of various functional groups related to HAp (Figure S4). The phase purity of HAp used in the current study was confirmed by XRD. It was found that the XRD pattern of HAp perfectly matched with the standard ICDD data card: 01-072-1243 (Figure S5).

**Figure 3:**
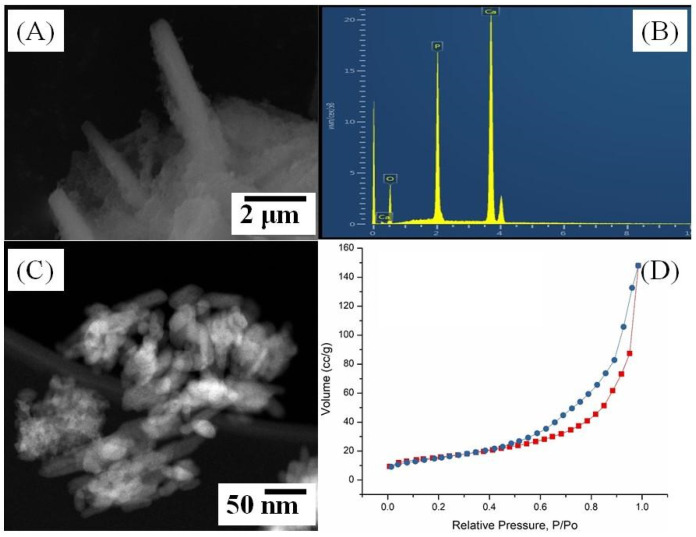
SEM micrograph (**A**), EDX spectrum (**B**), HAADF-STEM (**C**) image and nitrogen adsorption–desorption isotherm of commercially purchased HAp (**D**).

The surface morphology of commercially purchased HAp was studied by SEM (Figure 3A) and TEM (Figure 3C), showing that the HAp possesses rod-like morphology. The elemental analysis showed the characteristic peaks associated with calcium, oxygen, and phosphorus (Figure 3B) in a ratio corresponding to the composition of HAp.

For the HAp sample, the adsorption–desorption curve corresponds to type IV (Figure 3D) typical for mesoporous adsorbents [48]. The hysteresis loop corresponds to type H3 due to the absence of an obvious saturated adsorption platform at high pressure, which is typical for this material [49]. The average pore diameter was 2.9 nm, with a total mesopore volume of 0.237 cm^3^/g. The specific surface area, according to BET, is 55.2 m^2^/g.

### 3.3. FT-IR Characterization of PLA and 3D Printed Scaffolds

**Figure 4:**
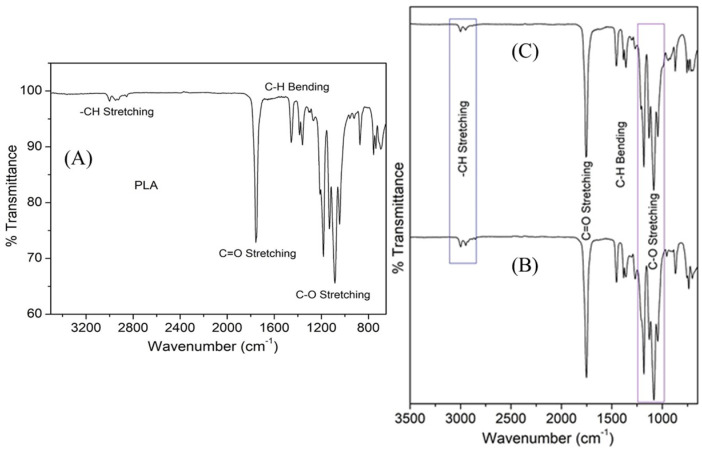
FT-IR spectra of commercially purchased PLA (**A**), PLA/HAp (**B**), and PLA/Wollastonite (**C**) composite.

FT-IR spectra of PLA are shown in Figure 4A. The band at 2998 cm^−1^ was related to asymmetric stretching of -CH_3_, whereas symmetric stretching vibration at 2952 cm^−1^ corresponds to the -CH_3_ group. C=O stretching band was noticed at 1756 cm^−1^. The band at 1456 cm^−1^ was due to asymmetric bending of -CH_3_, while symmetric bending vibration at 1359 cm^−1^ was associated with -CH_3_. Intense peaks ranging from 1045 cm^−1^ to 1182 cm^−1^ correspond to the C-O stretching vibration of esters, whereas the vibrations from 736 cm^−1^ to 871 cm^−1^ correspond to C-COO bands. This shows the crystalline nature of PLA. This observation was found to be similar to earlier reports [50].

The FT-IR spectra of composites (Figure 4B,C) were found to be similar to that of pure PLA. The potential reason for such observation was due to the occurrence of characteristic vibrational bands of hydroxyapatite and wollastonite at similar wavenumbers to that of PLA. The bands of wollastonite and HAp overlapped with the polymer. Moreover, minor shifting in the intensity of vibration bands was noticed. Zimina et al. (2020) also reported similar behavior in FT-IR spectra in their recent study [50].

**Figure 5:**
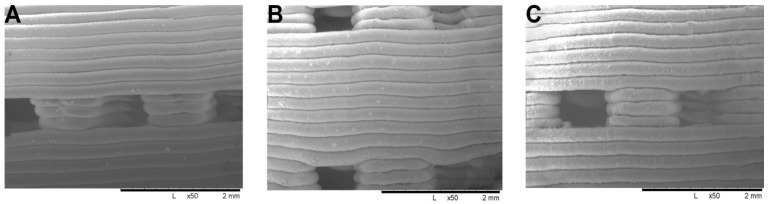
Structure of 3D printed PLA-based scaffolds: PLA (**A**); PLA/HAp (**B**); PLA/Wol (**C**), magnification ×50.

Scanning electron microscopy was used to analyze the microstructure of 3D printed scaffolds. Pore size decreased within 3D printing due to the thermal expansion and spreading of PLA, while pores formed interconnected structures through a network of channels. However, the average pore size was maintained at 600 μm (Figure 5).

### 3.4. Mechanical Testing of 3D Printed Scaffolds

**Figure 6:**
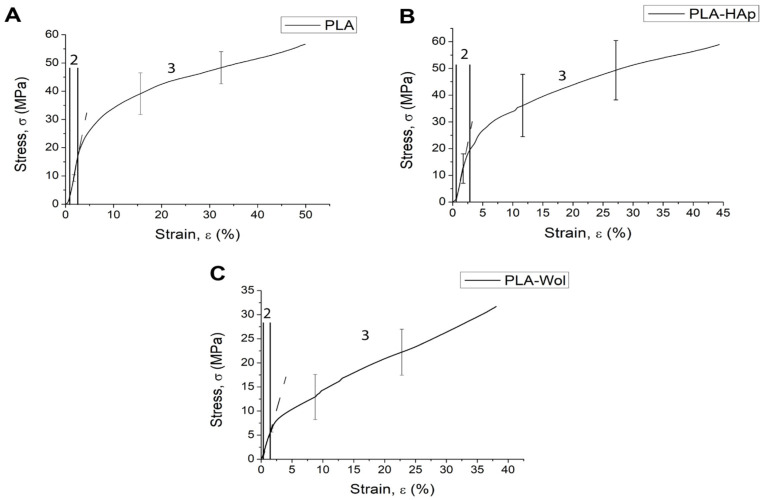
“Stress–strain” curve for PLA (**A**), PLA/HAp (**B**), PLA/Wol (**C**) scaffolds.

The stress–strain curve at the initial stage is typical for porous materials (Figure 6). Deformation of micropores and local deformations of the vertical walls of the specimen occurred with increasing load. Local deformations of vertical walls led to the propagation of multiple cracks and “folding” of the specimen. This is confirmed by the results of the Finite Element Analysis (FEA), where the maximum von Mises stresses were observed in the vertical walls of the pores, which led to the destruction of the structure (Figure S6). However, in this case, no “folding” of the specimens have yet been observed in the linear region.

The mechanical behavior of the specimens was directly described by the projection into three regions: linear strain region, nonlinear strain region, and yield region (Figure 6). The mechanical behavior of the specimens began to change in region two when the yield strength was reached, corresponding to 25 MPa for PLA, 21 MPa for PLA/HAp, and 7 MPa for PLA/Wol (Figure S7A). The Young’s modulus of PLA, PLA/HAp, and PLA/Wol was found to be 1814 MPa, 1764 MPa, and 575 MPa, respectively (Figure S7B).

The irreversible deformation of the samples was observed in region three, when the ultimate compressive strength limit was achieved, corresponding to 65 MPa for PLA, 59 MPa for PLA/HAp, and 32 MPa for PLA/Wol (Figure S8).

### 3.5. In Vitro Studies

Previously it has been reported that wettability is a key factor in the stimulation of cell adhesion and proliferation and osseointegration by a bone scaffold [51,52,53]. The addition of HAp and wollastonite to the PLA matrix significantly affected the surface properties of the samples. This observation can be compared with the illustrations shown in Figure S9. The addition of hydroxyapatite reduced the contact angle from (82 ± 3)° to (73 ± 5)°, and the incorporation of wollastonite decreased to (70 ± 6)°.

#### 3.5.1. Antibacterial Activity

**Figure 7:**
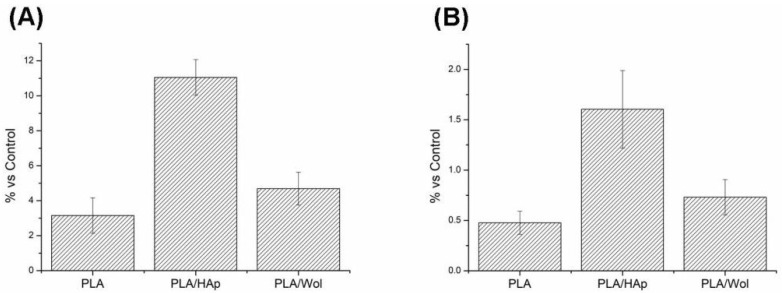
Fluorescence of live *E. coli* bacteria on the surface of samples (**A**) and the ratio of live and dead bacteria adhered to the surface (**B**).

*E. coli* is reported as one of the most dominant biofilms forming microorganisms in medical devices [54]. Thus, *E. coli* was used as a bacterial model for evaluating the stimulation of the adhesion of live bacteria on PLA-based samples. Syto 9 staining (Sigma, USA) was used to measure the intensity of live bacteria fluorescence on the PLA-based samples (Figure S10). Quantitative measurement of fluorescence showed that (Figure 7A) adhesion of bacteria on PLA was less than PLA/HAp and PLA/Wol due to the different hydrophilicity.

The obtained results indicated that the PLA stimulated adhesion only of 3 ± 1% of live bacteria. The addition of bioceramics slightly enhanced the adhesive properties of the samples in comparison with pure PLA: the PLA/HAp sample by five times (*p* < 0.05) and wollastonite by 2% (*p* > 0.05).

Antibacterial activity was studied according to the live/dead bacteria ratio adhered to the surface of PLA-based materials. The live and dead bacteria were examined by Syto 9 and propidium iodide (Figure S11). Quantitative measurement of fluorescence showed that (Figure 7B) the presence of wollastonite in the scaffold reduced the concentration of live bacteria on the surface of the sample as compared to PLA/HAp. Thus, wollastonite induced a bactericidal effect. This confirmed PLA/Wollastonite composite possesses an antibacterial property.

#### 3.5.2. MSCs Colonization

The colonization of mesenchymal cells on the surface of the scaffolds was evaluated after one week of deposition of cell suspension. The confluent cell monolayer was observed on the surface of all samples. At the same time, there was not a significant difference in the degree of cell colonization on the surface of the polylactide, polylactide/HAp, and polylactide/Wol scaffolds.

**Figure 8:**
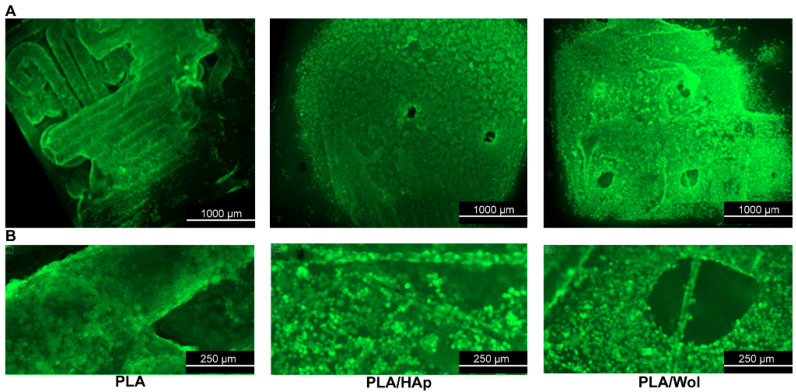
MSCs colonization of PLA-based sample surfaces: (**A**) ×2.5 magnification; (**B**) ×4 magnification.

The cell confluence on the surface of the scaffolds did not differ significantly from others (Figure 8). The MSCs covered the entire surface of the PLA, PLA/HAp, and PLA/Wol samples, being so close to each other that most of them had a rounded shape. It is obvious that this effect was the result of accelerated cell growth on the surface of these samples, not only on horizontal surfaces but also on vertically oriented details of the microrelief. Based on the recorded data, there was no evidence of a reliable effect of hydroxyapatite or wollastonite addition on the rate of cell colonization intensity.

## 4. Conclusions

This study demonstrated the low-temperature synthesis of wollastonite. The mechanical, structural, and biological properties of PLA containing wollastonite and HAp were evaluated and compared. PLA/Wol scaffolds revealed lower compressive strength as well as Young’s modulus as compared to PLA and PLA/HAp. The presence of wollastonite inhibited the bacteria biofilm formation, and MSCs were colonized on the surface. Thus, PLA/Wol scaffold can be a promising candidate for the development of submersible non-load orthopedical and maxillofacial implants for the prevention of implant-associated biofilms. These findings indicate wollastonite as an alternative filler of polymer-based composites for bone scaffold manufacturing.

## Supplementary Materials

The following supporting information can be downloaded at: https://www.mdpi.com/article/10.3390/polym14193932/s1, Figure S1: Specimens of 3D printed PLA (A), PLA/HAp 20% (B), and PLA/Wol 20% (C); Figure S2: Thermal behavior of wollastonite precursor; Figure S3: FT-IR spectra of combusted precursor (A), ball milled wollastonite (B), wollastonite calcined at 800 °C (C); Figure S4: FT-IR spectra of commercially purchased hydroxyapatite; Figure S5: XRD pattern of commercially purchased hydroxyapatite; Figure S6: Results of simulation mechanical test obtained by Finite Element Analysis (FEA) for PLA (A), PLA/HAp (B), PLA/Wol (C) 3D models; Figure S7: Ultimate Compressive strength (A) and Young’s modulus (B) for PLA, PLA/HAp, PLA/Wol scaffolds; Figure S8: Samples of 3D-printed PLA (A), PLA/HAp (B), and PLA/Wol (C) after static compression test; Figure S9: Measurement of contact angle on PLA, PLA/HAp, PLA/Wol; Figure S10: Fluorescence of live *E. coli* bacteria on the surface of samples after Syto 9 staining (Sigma, USA): PLA (A), PLA/HAp (B), and PLA/Wol (C); Figure S11: Comparative degree of adhesion of live (green) and dead (red) *E. coli* bacteria on the surface of polylactide samples of different composition: PLA (A), PLA/HAp (B), and PLA/Wol (C). Coloring with Live/Dead BacLight (Sigma, USA); Table S1: Print settings for PLA, PLA/HAp 20%, and PLA/Wol 20% composites.
