# Supplementary material for: Mechanical, Structural, and Biological Characteristics of Polylactide/Wollastonite 3D Printed Scaffolds
Source: Polymers (Basel). 2022 Sep 20;14(19):3932. doi: 10.3390/polym14193932 (PMC9572193; doi:10.3390/polym14193932)
Supplement: Supplementary file 1 [file polymers-14-03932-s001.zip › polymers-1901894-supplementary.pdf]

Supplementary Information

Mechanical, Structural, and Biological Characteristics of Polylactide/Wollastonite 3D Printed Scaffolds

Rajan Choudhary [1,2,3,*], Inna Bulygina [3,*], Vladislav Lvov [3], Anna Zimina [3], Sergey Zhirnov [3], Evgeny Kolesnikov [3], Denis Leybo, Natalya Anisimova [3,4], Mikhail Kiselevskiy [3,4], Maria Kirsanova [5], and Fedor Senatov [3]

[1] Rudolfs Cimdins Riga Biomaterials Innovations and Development Centre of RTU, Faculty of Materials Science and Applied Chemistry, Institute of General Chemical Engineering, Riga Technical University, Kipsala Street 6A, LV-1048 Riga, Latvia
[2] Baltic Biomaterials Centre of Excellence, Headquarters at Riga Technical University, Kipsala Street 6A, LV-1048 Riga, Latvia
[3] National University of Science and Technology "MISIS", 119049, Leninskiy pr. 4, Moscow, Russian Federation
[4] N. N. Blokhin National Medical Research Centre of oncology of the Health Ministry of Russia (N. N. Blokhin NMRCO), 115478, Kashirskoye sh. 24, Moscow, Russia
[5] Advanced Imaging Core Facility, Skolkovo Institute of Science and Technology, 3 Nobel Str., Moscow, 121205, Russia
* Correspondence: rajan.choudhary@rtu.lv (R.C.); ibulygina@misis.ru (I.B.)

Supplementary information includes:

Table S1

Figures S1–S11

Supplementary references

	PLA	PLA/HAp 20%	PLA/Wol 20%
Layer height	0.2 mm	0.2 mm	0.2 mm
Print speed*	20 mm/sec	20 mm/sec	17 mm/sec
Extruder temperature	195 ˚C	185 ˚C	165 ˚C
Bed temperature	65 ˚C	65 ˚C	55 ˚C
Extrusion multiplier	0.8	1	0.8
Nozzle diameter	0.4 mm	0.4 mm	0.4 mm

* - average print speed, including perimeters (interior and external), infill, bridges, etc.

Table S1. Print settings for PLA, PLA/HAp 20%, and PLA/Wol 20% composites. The settings presented in the table were optimized for each filament using PrusaSlicer v.2.3.1 software.

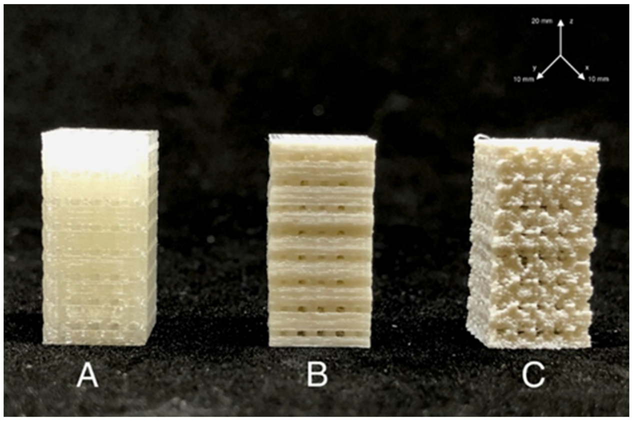

Figure S1. Specimens of 3D printed PLA (A), PLA/HAp 20% (B), and PLA/Wol 20% (C). Samples of rectangular cross-section with dimensions of 10x10x20 mm were prepared for mechanical testing according to ASTM D695.

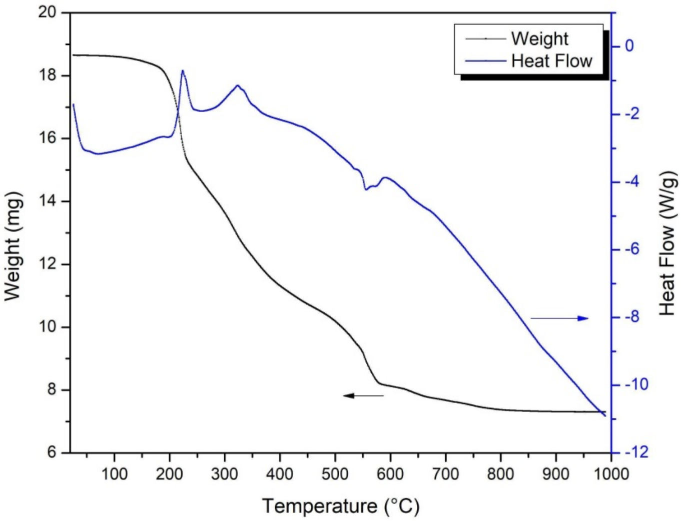

Figure S2. Thermal behavior of wollastonite precursor. The thermal behavior of the wollastonite precursor was analyzed from room temperature to 1000 °C. The major weight loss from the wollastonite thermogram was noticed in three steps. The first weight loss up to 240 °C was due to removal of ethanol and residual water from the dried powder. The second weight loss occurring between 240 °C to 460 °C was attributed to the decomposition of fuel as well as hydrated water molecules. The elimination of nitrate and residual organic moieties was observed from 460 °C to 650 °C. The majority of weight loss from the sample was noticed in these regions and after these events, a negligible weight loss was noticed. Moreover, the wollastonite dried precursor showed two endothermic and two exothermic peaks in the graph. The broad endothermic peak ranging from 50 °C to 200 °C is accounted for the dehydration of the sample. The exothermic peaks at 225 °C and 325 °C are assigned to the decomposition of fuel. The endothermic peak at 560 °C is related to the elimination of organic residual content.

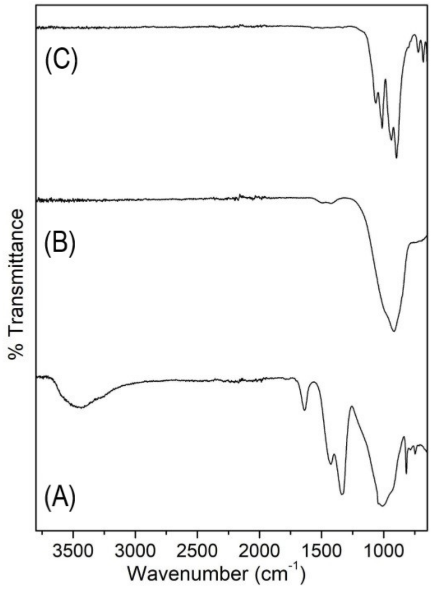

Figure S3. FT-IR spectra of combusted precursor (A), ball milled wollastonite (B), wollastonite calcined at 800 °C (C). The wollastonite precursor after combustion (Figure S3A) shows the stretching C-O vibration at 1430 cm^{-1} and bending C-O vibration at 815 cm^{-1} [1,2]. These functional groups were associated with the calcite phase of $CaCO_3$. The bending vibrations of water were noticed at 1637 cm^{-1} and the broad band at 3500 cm^{-1} is due to moisture absorption. The symmetric stretching and bending vibrations of Si-O were found at 1006 cm^{-1} and 746 cm^{-1}. The N-O symmetric stretch at 1334 cm^{-1} is due to the decomposition of residual organic moieties. The nitrate and calcite functional groups disappeared from the sample calcined at 700 °C and ball milled (Figure S3B). This shows their complete removal from the sample. A broad band was found to be associated with non-bridging oxygen Si-O-Ca bond [3]. When the sample was calcined at 800 °C for 2 hours (Figure S3C), a well-defined FT-IR spectra containing all the necessary functional groups related to wollastonite was obtained. The Si-O bending mode was noticed in the range of 680 cm^{-1} to 720 cm^{-1} whereas the stretching non-bridging oxygen vibration of Si-O-Ca was found at 896 cm^{-1} and 939 cm^{-1} respectively. The doublet Si-O-Si stretching was noticed at 1014 cm^{-1} and 1064 cm^{-1}. The presence of these functional groups confirms the preparation of wollastonite [1 (p. 4)].

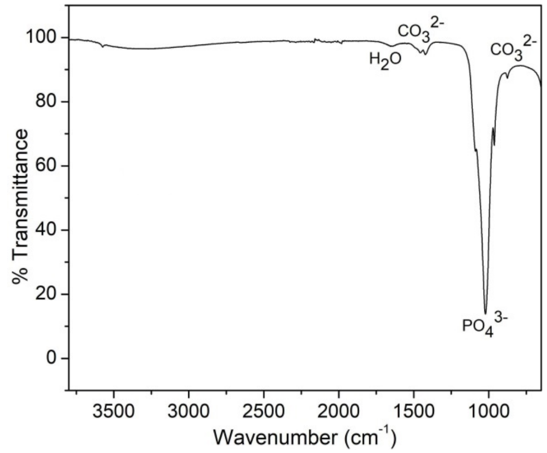

Figure S4. FT-IR spectra of commercially purchased hydroxyapatite. The stretching vibration bands at 962 cm^{-1}, 1022 cm^{-1}, and 1093 cm^{-1} shows the existence of phosphate group. The water absorption band was

detected at 1654 cm^{-1}. The asymmetric bending and stretching vibrations at 875 cm^{-1} and 1417 cm^{-1} corresponds to the carbonate group. This shows carbonate ion substitution at the phosphate ion site. These functional groups were found similar to an earlier finding [4].

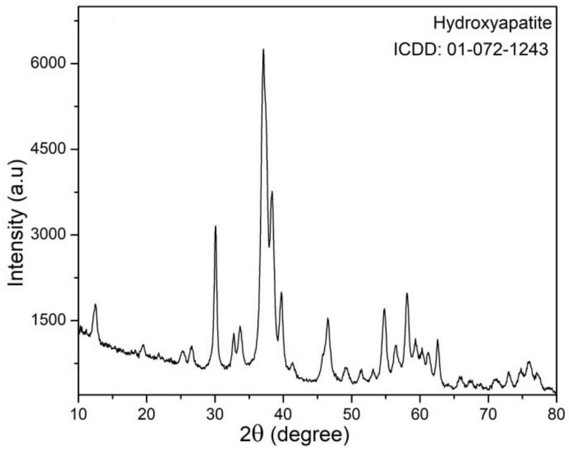

Figure S5. XRD pattern of commercially purchased hydroxyapatite. The phase purity of commercially purchased hydroxyapatite used in the current study was confirmed by XRD. It was found that the XRD pattern of HAp perfectly matched with the standard ICDD data card: 01-072-1243. Moreover, the lattice parameter values of the hydroxyapatite were found to be a= 9.4204 Å, b= 9.4204 Å, and c= 6.8839 Å.

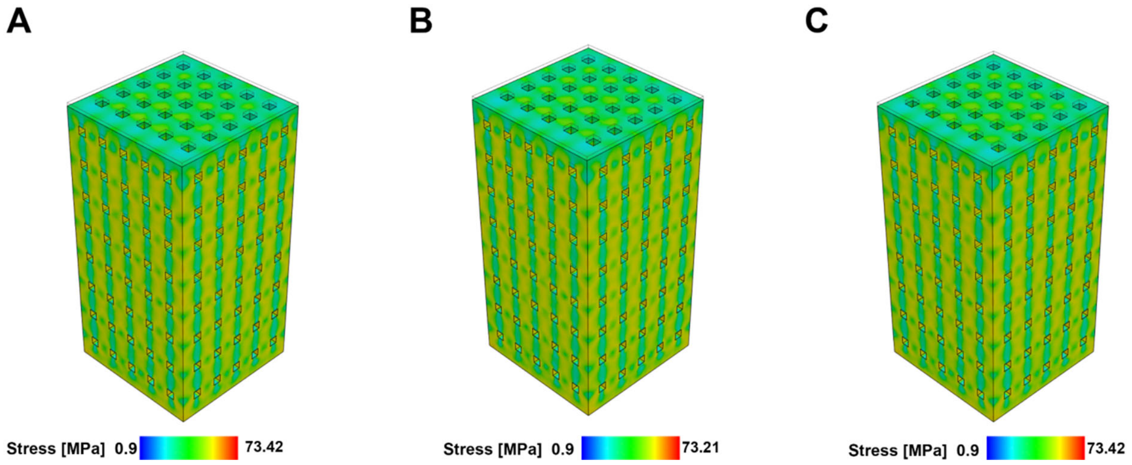

Figure S6. Results of simulation mechanical test obtained by Finite Element Analysis (FEA) for PLA (A), PLA/HAp (B), PLA/Wol (C) 3D models.

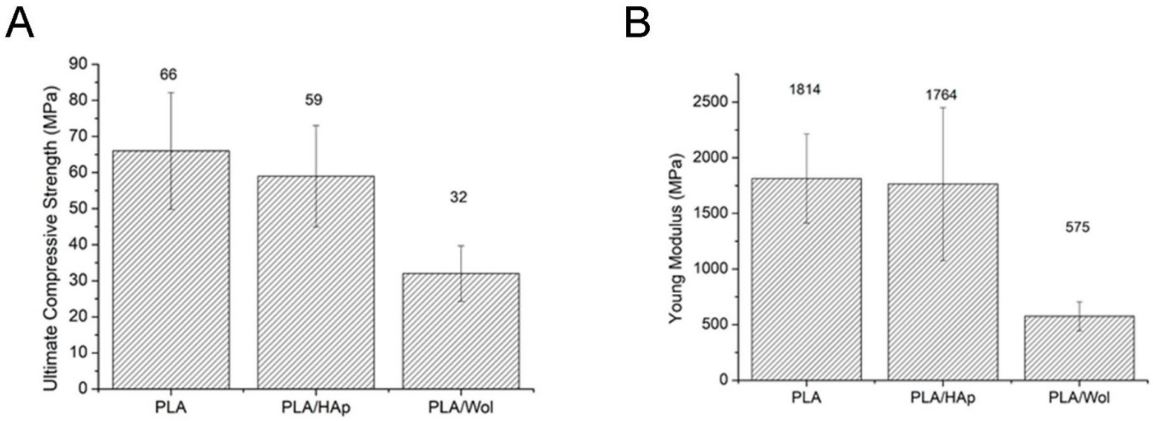

Figure S7. Ultimate Compressive strength (A) and Young's modulus (B) for PLA, PLA/HAp, PLA/Wol scaffolds.

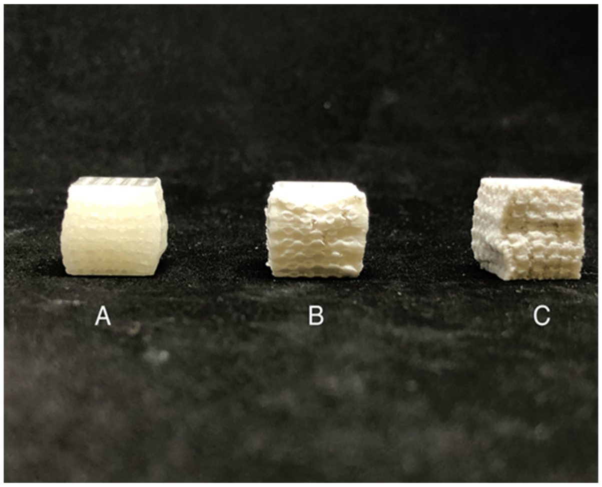

Figure S8. Samples of 3D-printed PLA (A), PLA/HAp (B), and PLA/Wol (C) after static compression test. Local deformations of vertical walls led to the propagation of multiple cracks and "folding" of the specimen.

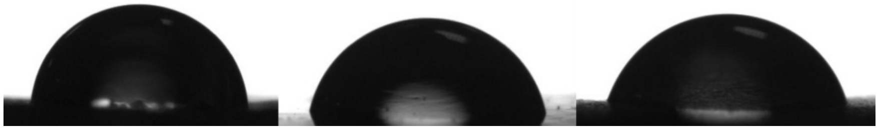

Figure S9. Measurement of contact angle on PLA, PLA/HAp, PLA/Wol. Contact angle of PLA was (82 ± 3)°, PLA/HAp – (73 ± 5)°, PLA/Wol – (70 ± 6)°.

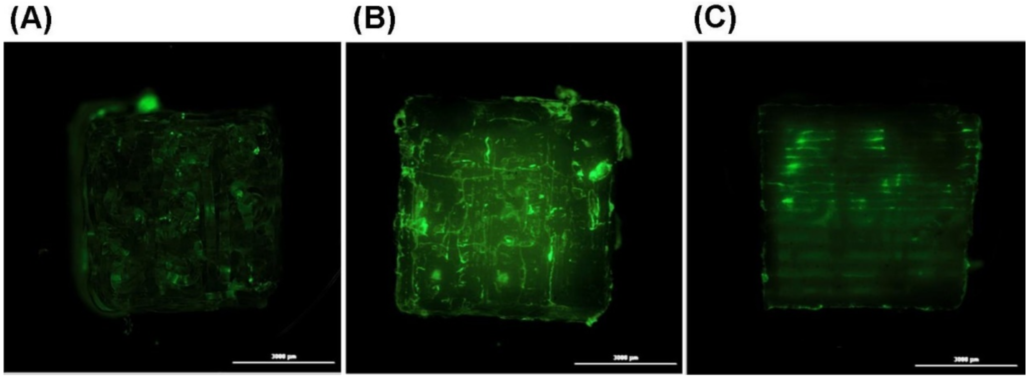

Figure S10. Fluorescence of live *E. coli* bacteria on the surface of samples after Syto 9 staining (Sigma, USA): PLA (A), PLA/HAp (B), and PLA/Wol (C).

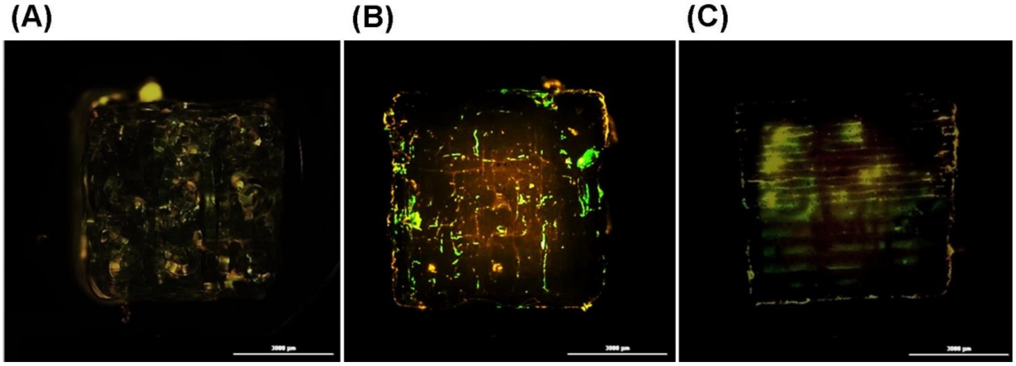

Figure S11. Comparative degree of adhesion of live (green) and dead (red) *E. coli* bacteria on the surface of polylactide samples of different composition: PLA (A), PLA/HAp (B), and PLA/Wol (C). Coloring with Live/Dead BacLight (Sigma, USA).

Supplementary references:

1. Choudhary, R.; Venkatraman, S.K.; Bulygina, I.; Senatov, F.; Kaloshkin, S.; Anisimova, N.; Kiselevskiy, M.; Knyazeva, M.; Kukui, D.; Walther, F.; et al. Biomineralization, Dissolution and Cellular Studies of Silicate Bioceramics Prepared from Eggshell and Rice Husk. Materials Science and Engineering: C 2021, 118, 111456, doi:10.1016/j.msec.2020.111456.
2. Anjaneyulu, U.; Sasikumar, S. Bioactive Nanocrystalline Wollastonite Synthesized by Sol–Gel Combustion Method by Using Eggshell Waste as Calcium Source. Bull Mater Sci 2014, 37, 207–212, doi:10.1007/s12034-014-0646-5.
3. Lakshmi, R.; Velmurugan, V.; Sasikumar, S. Preparation and Phase Evolution of Wollastonite by Sol-Gel Combustion Method Using Sucrose as the Fuel. Combustion Science and Technology 2013, 185, 1777–1785, doi:10.1080/00102202.2013.835308.
4. Zimina, A.; Senatov, F.; Choudhary, R.; Kolesnikov, E.; Anisimova, N.; Kiselevskiy, M.; Orlova, P.; Strukova, N.; Generalova, M.; Manskikh, V.; et al. Biocompatibility and Physico-Chemical Properties of Highly Porous PLA/HA Scaffolds for Bone Reconstruction. Polymers 2020, 12, 2938, doi:10.3390/polym12122938.
